# Transcutaneous electrical stimulation of somatic afferent nerves in the foot relieved symptoms related to postoperative bladder spasms

**DOI:** 10.1186/s12894-017-0248-9

**Published:** 2017-07-13

**Authors:** Chanjuan Zhang, Zhiying Xiao, Xiulin Zhang, Liqiang Guo, Wendong Sun, Changfeng Tai, Zhaoqun Jiang, Yuqiang Liu

**Affiliations:** 1 Department of Urology, The Second Hospital of Shandong University, 247 Beiyuan Street, Jinan, 250033 China; 2 Department of Urology, University of Pittsburgh, Pittsburgh, PA USA; 3 Department of Pharmacology and Chemical Biology, University of Pittsburgh, Pittsburgh, PA 15213 USA

**Keywords:** Transcutaneous electrical stimulation, Afferent nerve, Bladder spasm

## Abstract

**Background:**

Bladder spasm is a common side effect of urological surgery. Main treatment modalities include opioids or anticholinergic medication; however, bladder spasms still occur even after these interventions. Recent studies indicate that transcutaneous stimulation of the foot can result in 50% increase in bladder capacity in healthy adults, and inhibit bladder detrusor overactivity in spinal cord injured patients. In this study, we examined the effects of transcutaneous electrical stimulation of the foot on bladder spasms related symptoms.

**Methods:**

Sixty-six male patients who underwent prostate or bladder surgeries due to benign prostatic hyperplasia or bladder diseases were randomly divided into two groups: the control group (*n* = 36) and the treatment group (*n* = 30). The control group received the routine postoperative care. The treatment group received daily transcutaneous electrical stimulation of the foot during 3 days after surgery; each time lasted for 60 min. All patients were evaluated by the Visual Analogue Scale for pain sensation, frequency of bladder spasm episodes, and a total score of bladder spasms symptoms.

**Results:**

In the control group, the patients with bladder surgery had a higher Visual Analogue Scale score than patients with prostate surgery (*P* = 0.024). In both treatment and control groups, the Visual Analogue Scale score, spasm frequency, and total score of bladder spasm symptoms decreased from day 1 to day 3 (*P* <0.001). The Visual Analogue Scale score at day 2, total score of bladder spasm symptoms at day 2 and day 3 were significantly lower in the treatment group than in the control group (*P* <0.05).

**Conclusion:**

These results provided preliminary evidence suggesting beneficial effects of stimulating somatic afferent nerves in the foot on postoperative bladder spasms.

**Trial registration:**

The study was registered with Chinese Clinical Trial Registry on June 13 2016 (http://www.chictr.org.cn/) (Identifier: ChiCTR-INR-16008635)

## Background

Postoperative bladder spasms are involuntary movements of the detrusor muscle that can cause a sudden onset of pain in the region of bladder, a sensation of urgency to void, intermittent abdominal cramps, perineal pain, and/or urinary leakage around a urethral catheter after surgical procedures [1–3]. The intermittent bladder pain is usually short, lasting 30 s or more with an interval about several minutes or hours. Bladder spasm is a common side effect of urological surgery; it could result in not only postoperative pain but also hemorrhage and therefore may prolong bladder recovery. Current treatment modalities include opioids [4], anticholinergic medication [5], bladder smooth muscle relaxants [3] and sedation [6]. However, bladder spasms still occur even though the patients are treated by these interventions.

Transcutaneous electrical nerve stimulation is a non-pharmacological and non-invasive method that delivers electrical pulses to the nerve via skin surface electrodes and widely used for pain relief [7–9]. Recent studies indicate that transcutaneous stimulation of the foot can result in more than 50% increase in bladder capacity in healthy adults [10], and inhibit bladder detrusor overactivity in spinal cord-injured patients [11]. Therefore, we hypothesize that transcutaneous electrical stimulation of the foot might partially or completely relieve the symptoms caused by postoperative bladder spasms. To test this hypothesis, this study examined the effects of electrical stimulation of the foot in patients who underwent surgical procedures for benign prostate hyperplasia (BPH) or bladder diseases.

## Methods

This prospective and randomly controlled study was reviewed and approved by the ethical committee of the second hospital of Shandong University in China(20150024), and was conducted in the urology department of the hospital. The written informed consent was obtained from each participant prior to enrollment and the study was registered with Chinese Clinical Trial Registry (http://www.chictr.org.cn/) (Identifier: ChiCTR-INR-16008635).

### Patients and study design

Male patients who underwent operations for BPH (*n* = 41) or bladder diseases (*n* = 25) were enrolled between June 2016 and August 2016. Patients mean age was 64.5 years ranged from 54 to 79 years. The inclusion criteria include patients: (1) with BPH diagnosed preoperatively by urosonography, prostate specific antigen, digital rectal examination and urodynamic tests, or with bladder disease confirmed preoperatively by cystoscopy; (2) can complete the three times stimulation without missing anyday; (3) without neurogenic bladder, urinary tract infection, urinary incontinence, and/or cardiac pacemaker. In 41 patients with BPH, transurethral holmium laser enucleation of the prostate were performed by one experienced surgeon and his assistants under epidural anesthesia, In 25 patients with bladder diseases, transurethral vaporization of bladder lesions with laser or transurethral electro-resection of bladder lesions was performed under epidural anesthesia by the same experienced surgeon. All patients had indwelling catheters (22 F) during the study period, and the anticholinergics or antibiotics were not applied.

**Fig. 1:**
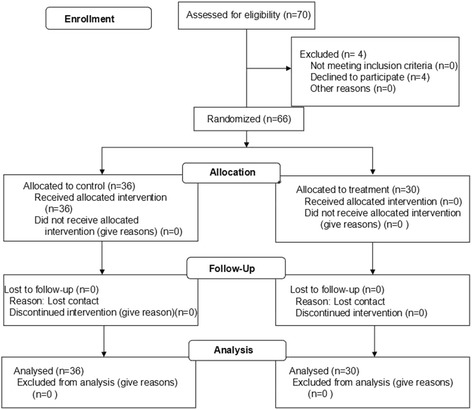
Consolidated standards of reporting trials flow chart for the trial

The current study was reported according to CONSORT guidelines (Fig. 1). Initially, seventy patients were enrolled in this study, 4 patients declined to participate, 66 patients met the inclusion criterion, and were randomly divided into two groups: the control group (*n* = 36) and the treatment group (*n* = 30) following a computer generated randomization list. A simple random allocation sequence was generated and concealed by a trained nurse. There were 22 patients who underwent prostate surgery and 14 patients had bladder surgery in control group, while in the treatment group, the numbers were 19 and 11, respectively.

The control group patients received routine postoperative care including health education for diet plan, postoperative body positioning and catheterization, and management of adverse effects after surgery. For patients in treatment group all the interventions are the same as control group except they underwent daily electrical stimulation of the foot for 3 days after surgery; each time lasted for 60 min. The first stimulation was given at 12 h postoperatively and repeated at day 2 and 3, respectively. After each stimulation, patients were evaluated by the Visual Analogue Scale (VAS, from 0 to 10) for pain, spasm frequency and total score of bladder spasms symptoms. Data were collected at the same time points in control group and treatment group, i.e. 12, 36 and 60 h after surgery in the patient bedside.

### Electrical stimulation protocol

**Fig. 2:**
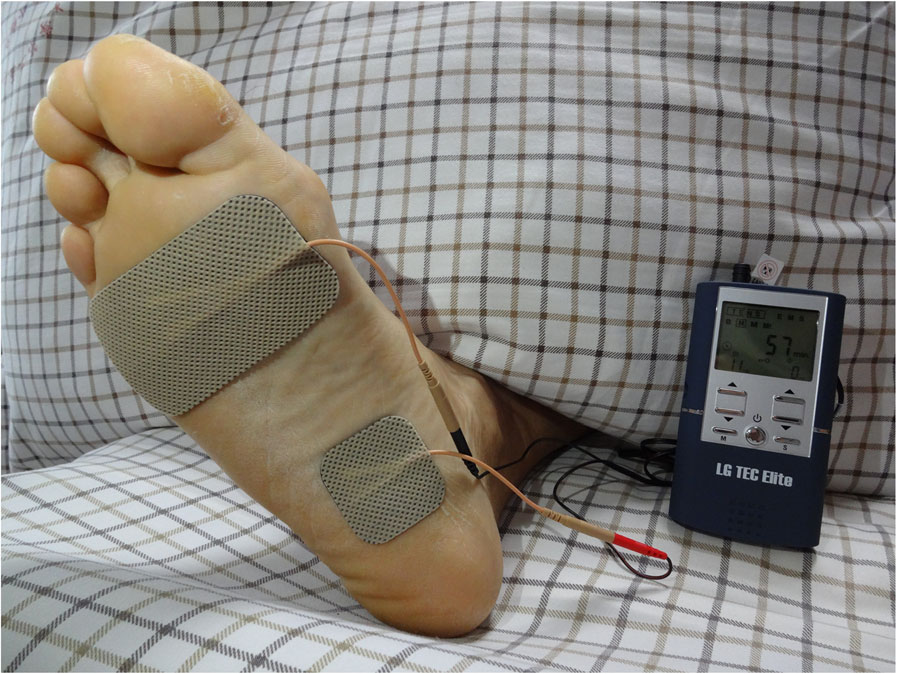
The position of two skin surface electrodes and the connection with the stimulator. A large cathodal electrode (2 × 3.5 in.) was placed on the front of the foot and a small anodal electrode (2 × 2 in.) was placed between the inner foot arch and the heel. A written consent was obtained from the patient for publication of the image in the journal

We referred a report from Dr. Chen for the stimulation method [10]. Briefly, two skin surface electrodes (LGMedSupply, Cherry Hill, New Jersey, USA) were placed over the bottom of foot and connected to a TEC Elite transcutaneous electrical nerve stimulator (LGMedSupply, New Jersey, USA) which provided constant current, rectangular pulses of 5 Hz frequency and 0.2 millisecond pulse width (Fig. 2). The stimulation threshold was defined as the minimal intensity for inducing toe twitching. Stimulation intensity was then increased to the maximal level (ranging from 35 to 105 mA, or 2-6 times of the stimulation threshold) comfortable to the subject for the entire 60 min stimulation. The stimulation was conducted in the hospital, and was finished by one tester who was also responsible for data collection.

### Assessment of bladder spasm and pain

Visual Analogue Scale (VAS, from 0 to 10) for pain was used to measure bladder spasm-related pain. In addition to pain sensation, patients with bladder spasm usually have other presentations including urgency, urinary leakage. To comprehensively assess the symptom severity of bladder spasm, a questionnaire, which was based on the answers to five questions including urination feeling, urgency incontinence, spasm episodes, bladder pain, and urinary leakage around catheter, was developed and used in this study. The total score of bladder spasm symptoms was calculated by adding the five sub-scores as shown in Table 1.

**Table 1:** The total score of bladder spasms symptoms was calculated by adding scores from every sub-symptom

Score	0	2	4	6
Sub-symptoms				
Urgency	Never	Mild	Moderate	Severe
Bladder pain	Never	<30 min/day	30-60 min/day	>60 min/day
Urgent incontinence	Never	1/day	2-4/day	>4/day
Urinary leakage around catheter	Never	Rarely	Sometimes	Often
Spasm episode	<2/day	2-5/day	5-7/day	>7/day

### Primary and secondary outcomes

The primary outcomes were bladder spasm frequency and VAS score, the secondary outcome was the total score of spasm.

### Statistical analysis

All data were expressed as mean ± SD. The Sigmaplot program was used for the data analysis. The sample size was calculated to be at least 30 patients in each group with α = 0.05, β = 0.10, a desired statistical power level of 90% and 60% reduction of spasm episode at day 2 in treatment group relative to control group. In both control and treatment groups, the time course of spasm severity (time effect), the effects of electrical stimulation and interactions between stimulation and time were analyzed by two-tailed repeated measures ANOVA followed by Holm-Sidak post hoc tests to detect the statistical significance (*P* <0.05).

## Results

The final number of patients analyzed for control group was 36, and it was 30 for treatment group (Fig. 1). There was no significant difference in subject distribution of the two different surgeries (prostate/bladder) between control and treatment groups (22/14 for control and 19/11 for treatment, χ^2^ test, *P* >0.05). In the control group, patients with bladder surgery had higher VAS score than those with prostate surgery (*P* = 0.024), spasm frequency and total score of bladder spasms symptoms were also higher for bladder surgery but did not reach significance (*P* >0.05) (Table 2).

**Table 2:** Comparison of VAS score, spasm frequency and total symptom score between prostate and bladder surgery in control group

	Prostate surgery (*n* = 22)	Bladder surgery (*n* = 14)	P
VAS score			
Day 1	3.76 ± 0.42	5.23 ± 0.47	0.014
Day 2	3.53 ± 0.44	4.54 ± 0.54	0.031
Day 3	1.94 ± 0.36	3.00 ± 0.65	0.027
Spasm episode			
Day 1	1.18 ± 0.42	2.00 ± 0.55	>0.05
Day 2	1.18 ± 0.42	2.15 ± 0.53	>0.05
Day 3	0.47 ± 0.36	0.92 ± 0.49	>0.05
Total score			
Day 1	9.06 ± 1.59	11.54 ± 2.20	>0.05
Day 2	8.59 ± 1.66	12.00 ± 2.34	>0.05
Day 3	4.71 ± 1.31	6.62 ± 2.23	>0.05

In both treatment and control groups, the VAS score (*P* <0.001), the spasms frequency (*P* = 0.003), and total score of bladder spasms (*P* <0.001) symptoms are all significantly decreased from day 1 to 3 (Table 3). There was no interaction between foot stimulation and time in treatment group for VAS (*P* = 0.194), bladder spasms (*P* = 0.418), and total score of bladder spasms (*P* = 0.864).

**Table 3:** Comparison of VAS score, spasm frequency and total score between control and treatment groups

	Control group (n = 36)	Treatment group (n = 30)	P
VAS score			
Day 1	4.48 ± 0.37	4.12 ± 0.37	>0.05
Day 2	3.85 ± 0.38	2.85 ± 0.23	0.027
Day 3	2.26 ± 0.38	1.38 ± 0.21	>0.05
Spasm episode			
Day 1	1.63 ± 0.37	1.51 ± 0.34	>0.05
Day 2	1.48 ± 0.36	0.38 ± 0.16	0.065
Day 3	0.59 ± 0.32	0.08 ± 0.08	0.086
Total score			
Day 1	10.30 ± 1.44	11.38 ± 1.59	>0.05
Day 2	9.41 ± 1.47	5.54 ± 0.75	0.020
Day 3	5.26 ± 1.30	2.15 ± 0.52	0.045

Our data revealed a lower VAS score in treatment group than control group on day 2 (*P* = 0.027, Table 3). A lower total score of bladder spasm symptoms in treatment group was revealed at day 2 (*P* = 0.020) and day 3 (*P* = 0.045) when compared to control group (Table 3). Patients who received foot stimulation tended to have less bladder spasm episodes than those in control group on day 2 and day 3, but the difference did not reach significant level (*P* = 0.065 and *P* = 0.086, respectively) (Table 3).

## Discussion

The management modalities for post operative bladder spasms include opioids [4], anticholinergic medication [5], bladder smooth muscle relaxants [3], sedation [6] and anaesthesia [1]. This study examined the effects of transcutaneous foot stimulation on patients with bladder spasms after prostate or bladder surgeries. It showed that foot stimulation could significantly reduce pain sensation and alleviate the symptoms of bladder spasms. These results provided preliminary evidence suggesting beneficial effects of stimulating somatic afferent nerves in the foot after bladder or prostate surgeries.

Previous studies showed that bladder and posterior urethral injuries associated with invasive procedures, postoperative catheterization, and bladder irrigation could induce involuntary contraction of detrusor muscle [1, 2, 12, 13]. In consistent with these reports bladder spasms were noticed in patients who underwent resection of the prostate or bladder lesions in our study. The mechanism underlying bladder spasm still remains unclear. It is well known that bladder mucosa, especially at the trigone is extremely sensitive to temperature, pressure and mechanical stimulation [14]. A higher VAS score was found in patients after bladder surgery than those after prostate surgery (Table 2), suggesting that bladder surgeries may result in more irritation of the bladder mucosa and trigone than prostate surgeries. There was a similar distribution of the bladder and prostate patients in the control and treatment groups. Therefore, the surgical effects on bladder spasm should be comparable between the two groups.

To our knowledge, there are no internationally recognized criteria for assessing bladder spasm. Currently, spasm frequency and VAS score are the most commonly used criteria to evaluate bladder spasm [1–3]. In this study, we combined these two parameters together with urination feeling, urgent incontinence and urinary leakage around catheter in order to measure the symptom severity of bladder spasm (Table 1). The total score of bladder spasm symptoms provided more comprehensive information than VAS score or spasm frequency alone, which could help clinicians better understand and manage postoperative bladder spasms.

The effects of foot stimulation was only observed on days 2 and 3 but not on day 1, indicating that repeated stimulation might be required in order to accumulate the therapeutic effects. Meanwhile, the stimulation was only applied 1 time/day but the effects seem sustained during the whole day, indicating that the stimulation must have induced post-stimulation effects. The post-stimulation inhibitory effects on bladder spasms are in agreement with previous studies showing that a post-stimulation inhibition of the micturition reflex can be induced by stimulation of the foot or tibial nerve in rats [15] and cat [16, 17]. In both treatment and control groups, the VAS score, spasm frequency, and total score of bladder spasm symptoms decreased from day 1 to day 3 (Table 3), which might be due to the natural healing process postoperatively.

Even though we did not know exactly which nerves were activated by foot stimulation, it is highly likely that it activates afferent axons in the lateral and medial plantar nerves of the foot, because the 2 skin surface electrodes were placed along the passage of these nerves (Fig. 2). A large body of animal studies indicated that tibial nerve neuromodulation inhibited bladder overactivity by activating spinal inhibitory neurotransmitters (opioid, gamma aminobutyric acid) and metabolic glutamate receptor 3 [16, 18, 19]. Inhibition of bladder spasm in the present study may also involve these inhibitory neurotransmitters. The activation of sensory nerves at foot might induce the release of inhibitory neurotransmitters that in turn produce an inhibitory interaction between somatic peripheral neuropathway and autonomic micturition reflex to suppress bladder hyperactivity and pain.

Neuromodulation is widely used as an alternative approach for treatment of bladder dysfunctions [18, 20]. Sacral neuromodulation is the most effective method, but it is invasive and requires high cost. Tibial neuromodulation is minimally invasive, that involves inserting a needle electrode near the ankle to stimulate the tibial nerve, there were no reports indicate the beneficial effects of tibial nerve stimulation on bladder spasm, however, it was proved as efficacious as antimuscarinic drugs for many bladder dysfunctions such as detrusor overactivity [21], a full-scale randomized trial (RCT) conducted in older adults in residential care homes suggested transcutaneous tibial nerve stimulation (PTNS) could reduce the number of episodes of urinary and fecal incontinence [22]; another RCT study conducted on overactive bladder patients demonstrated 12 month weekly PTNS had effectiveness on overactive bladder symptom improvement [23]. There are several advantages of foot stimulation used in this study over tibial neuromodulation: compeletely noinvasive, without adverse effects and can be conducted by patients themselves.

It should be noted that there are some limitations in the present study such as the absence of placebo controls. It is hard to design a placebo control for foot stimulation, since managements such as simply turning off the stimulation would be easily noticed by the patient.

## Conclusion

In summary, electrical foot stimulation could relieve the symptoms of bladder spasms and pain after BPH or bladder surgeries. Since foot stimulation is non-invasive, convenient for patients to use, and has no side effect, it could be considered as an additional modality for the control of postoperative bladder spasms and/or pain.

## Abbreviations

BPH: Benign prostate hyperplasia
VAS: Visual analogue scale
